# SARS-CoV-2 in dialysis patients and the impact of vaccination

**DOI:** 10.1186/s12882-022-02940-2

**Published:** 2022-09-21

**Authors:** Louise Rachel Moore, Noor Al-Jaddou, Harsha Wodeyar, Asheesh Sharma, Michael Schulz, Anirudh Rao, Kottarathil Abraham

**Affiliations:** 1 Liverpool University Hospitals NHS FT, Liverpool, UK; 2 Nephrology Department, Liverpool University Hospitals NHS Foundation Trust, Liverpool, UK

**Keywords:** Dialysis patients, Efficacy, SARS-CoV-2, Vaccination

## Abstract

**Background:**

In centre haemodialysis (ICHD) patients have been identified as high risk of contracting Severe Acute Respiratory Syndrome Coronavirus 2 (SARS-CoV-2) infection due to frequent healthcare contact and poor innate and adaptive immunity. Our ICHD patients were offered immunisation from January 2021. We aimed to assess outcomes following SARS-CoV-2 infection and report on the effect of vaccination in our ICHD patients.

**Methods:**

Demographics, SARS-CoV-2 status, hospitalisation, mortality and vaccination status were analysed. From 11^th^ March 2020 to 31^st^ March 2021, 662 ICHD patients were included in the study and these patients were then followed up until 31^st^ August 2021.

**Results:**

SARS-CoV-2 infection occurred in 28.4% with 51.1% of them requiring hospitalisation in contrast to community infection rates of 13.9% and hospitalisation of 9.0%. 28-day mortality was 19.2% in comparison to 1.9% of the community. Mortality increased to 34.0% over the study period. Mortality over the study period was 1.8 times in infected patients (HR 1.81 (1.32–2.49) *P* < 0.001) despite adjustment for age, gender and ethnicity. 91.3% of ICHD patients have now received both doses of SARS-CoV-2 vaccinations.

**Conclusions:**

ICHD patients are at increased risk of acquiring SARS-CoV-2, with increased rates of hospitalisation and mortality. The increased mortality extends well beyond the 28 days post-infection and persists in those who have recovered. Peaks and troughs in infection rates mirrored community trends. Preliminary data indicates that the SARS-CoV-2 vaccination provides protection to ICHD patients, with ICHD case rates now comparable to that of the local population.

## Background

Dialysis patients have been identified to be at high risk of contracting Severe Acute Respiratory Syndrome Coronavirus 2 (SARS-CoV-2) infection due to various factors such as poor immunity and frequent healthcare contact [1]. Higher infection rates are also associated with higher mortality rates in the in-centre haemodialysis (ICHD) cohort [2, 3]. Given that these patients must attend healthcare settings for haemodialysis, they cannot isolate themselves effectively [2]. While infection control measures help reduce the risk of contracting SARS-CoV-2 [4]; vaccination has been advocated to provide further protection. International consensus has highlighted the need for ICHD patients to be prioritised for vaccination [5]. In contrast to the general population in whom both the BNT162b2 (Pfizer BioNTech) and the ChAdOx1 (Astra Zeneca) vaccines have proved effective [6, 7], very little is known whether such a strategy translates into reduced infection or transmission rates in the ICHD population. A nephrology department led vaccination programme was introduced in January 2021 whereby the ICHD population were administered the ChAdOx1 vaccines unless they had already received the vaccination in primary care, vaccination was contraindicated or the patient refused.

The study aimed to assess outcomes in ICHD patients relating to hospitalisation and mortality following SARS-CoV-2 infection, comparing this to community data and to report the roll out and effectiveness of the vaccination programme in Liverpool.

## Methods

This was a prospective cohort study.

### Study setting

Liverpool is a large city in the North West of England, UK. Liverpool local authority is the third most deprived of 317 local authority areas across England including measures of health deprivation and disability [8]. The majority of Liverpool's population is White (84.8% White British, 1.4% White Irish and 2.6% White other), with 4.2% being Asian/Asian British, 2.6% Black/African/Caribbean/Black British, 2.5% mixed ethnicity and 1.8% of other ethnicities [9].

Liverpool University Foundation Hospital Trust runs a hub and spoke model for nephrology care with the two main nephrology centres based at Royal Liverpool University Hospital and Aintree University Hospital in Liverpool. Nephrology care is coordinated in the city with dialysis units across the region with two hub dialysis units and seven satellite units predominately based within Merseyside, with the Warrington and Halton units based in Cheshire.

### Study population

ICHD patients whose care was delivered by Liverpool University Foundation Hospital Trust (LUFHT) from 11^th^ March 2020 to 31^st^ March 2021 were included in the study and then followed up until 31^st^ August 2021. All patients who had received ICHD at some point during the study entry period were included. Royal Liverpool University Hospital and Aintree University Hospital house one hub unit on each hospital site, two in total and typically have patients of higher co-morbid burden and functional dependence. The department also operates seven satellite dialysis units.

We defined patients with a diagnosis of SARS-CoV-2 infection as those who tested positive for infection by reverse transcription − polymerase chain reaction (RT-PCR) assay for SARS-CoV-2 on a nasopharyngeal swab. Up until 17^th^ January 2021, SARS-CoV-2 testing was performed in those with symptoms and those who had known contact with a SARS-CoV-2 patient. From 18^th^ January 2021, LUFHT introduced weekly SARS-CoV-2 testing in all ICHD patients.

Exclusion criteria:

- Home haemodialysis patients
- Peritoneal dialysis patients
- Patients who required inpatient dialysis for acute kidney injury that recovered within 90 days.

### The introduction of the ICHD vaccine programme

In the UK, ICHD patients were recognised as being at high risk of SARS-CoV-2 infection and associated mortality and morbidity given their comorbidities and inability to effectively isolate having to attend healthcare settings thrice weekly for dialysis [10]. These observations underpinned the UK Renal Association position that ICHD patients should be prioritised for vaccination by the Joint Committee of Vaccination and Immunisation (JCVI) [10]. Furthermore, it was recommended that vaccination is undertaken at dialysis units, as this is where patients access the majority of their care. The national protocol for vaccine administration was adapted for local use [11]. All our ICHD patients were offered the first dose of the ChAdOx1 vaccination from January 2021 and the second dose was offered approximately twelve weeks later. The vaccination programme roll out was over a several week period, targeting the various dialysis units, whilst the patients were receiving haemodialysis.

### Data collection

The first case of SARS-CoV-2 infection recorded in the UK was towards the end of January 2020. Data from ICHD patients was collected from March 11^th^ 2020 (the start of the SARS-CoV-2 pandemic as declared by the World Health Organisation) [12] until approximately six months after the cohort were offered their first dose of the vaccine (31^st^ August 2021). Data was obtained from hospital electronic records and general practice (GP) summary care records. Baseline data included patient demographics including age at study entry, gender and ethnicity. SARS-CoV-2 vaccination status including date of vaccine administered and type of vaccine (BNT162b2, ChAdOx1) given were recorded, SARS-CoV-2 infection including date of positive RT-PCR swab, hospitalisation including dates of admission, WHO clinical progression score [13] and outcome were recorded. Vaccination data was collected from primary care records and our in-centre vaccination records. SARS-CoV-2 related mortality was classified as death within 28 days of positive RT-PCR swab [14]. Current mortality status (alive or dead) was captured. Patients were censored for modality change, move out of area or death. The vaccination and follow up data collection was prospective observational data. Data collection prior to the rollout of the vaccination programme was retrospective.

During the study period, there have been shifts in community transmission rates that have triggered nationwide closures of businesses, universities and schools, alongside measures for social distancing. These interventions have helped control rising infection rates, described in two waves in England and three in the Merseyside region. To adjust for the confounding impact of these social measures, government data on regional community infection, hospitalisation and mortality rates as well as vaccine uptake were also accessed over the same period on a month by month basis. Regional data was available from government sources by local authority [15]. The six local authorities that cover our patient population are Liverpool, Knowsley, Sefton, St Helens, Warrington, and Halton. Data on daily SARS-CoV-2 cases and deaths were collected and totalled to give monthly case and death numbers for our region. Daily hospital admission data for the hospital trusts within these six local authorities (Liverpool University Hospital Foundation Trust, Liverpool Women's Hospital, Liverpool Heart and Chest Hospital, Warrington and Halton Hospital Trust, St Helens and Knowsley Hospitals, Southport and Ormskirk Hospitals) were collected and totalled to calculate the daily hospital admission number for our region. A population estimate for the six local authorities was obtained from the Office of National Statistics website at 1 445 323 from mid-2019 [16]. Mortality rates over the previous 5 years in our ICHD population were used to calculate the expected number of deaths in this multimorbid group and from that, the excess deaths during the pandemic. SARS-CoV-2 vaccination data was collected for our ICHD patients and compared to the vaccination uptake data in the age 12 + population of Liverpool local authority [15].

### Statistical analysis

Summary statistics were produced using frequencies and proportions for categorical variables and means, standard deviations, medians, and ranges for numeric variables. The two cohorts (SARS-CoV-2 positive and negative) were compared using the chi-square test for categorical data, one-way analysis of variance for normally distributed numeric data, and the Mann–Whitney test for skewed numeric data.

A logistic regression model was used to identify variables that were associated with the risk of SARS-CoV-2 infection. We considered in the models the code 1 = SARS-CoV-2 positive and 0 = SARS-CoV-2 negative. Univariable logistic regression models were run for each of the following explanatory variables of known clinical importance: age, gender, ethnicity and dialysis location (hub versus satellite dialysis units).

A Cox proportional hazards regression model was used to compare all-cause mortality in the ICHD patients 18 months from the start of the pandemic. Univariable cox-regression models were run for each of the following explanatory variables of known clinical importance: age, gender and ethnicity.

The incidence rate of SARS-CoV-2 infection was calculated by dividing patients with infection each month by the patients at risk expressed as a percentage in the ICHD patients in Liverpool. As the risk of SARS-CoV-2 reinfection is uncommon during the initial 90 days after symptom onset of the primary infection; these patients were excluded from at-risk group (denominator). A similar calculation was performed for the community. Calculating the incidence rate of SARS-CoV-2 infection in the ICHD patients and community allowed direct comparison.

The ICHD vaccination rates were calculated every month by the number of patients vaccinated that month divided by ICHD patients alive that month, expressed as a percentage. This was reported as a cumulative rate.

To calculate the burden of SARS-CoV-2 infections before and after the 1^st^ dose of the SARS-CoV-2 vaccine the pre and post 1^st^ dose vaccination cohort was defined. Patients in the pre-vaccination cohort should have received the first vaccine and entered the study prior to receiving the first SARS-CoV-2 vaccination. The post-vaccination cohort should have received the first vaccine and exited the study after receiving the first SARS-CoV-2 vaccination. At risk period was calculated for the patients in each of the cohorts. For the pre-vaccine cohort by subtracting the date of the first vaccine from the study start date and for the post-vaccine cohort by subtracting the study end date from the date of the first vaccine. For patients who contracted SARS-CoV-2 90 days was subtracted from the risk period as reinfection is unlikely within 90 days of infection [17].

All analyses were performed using Stata v13.1 (College Station, Tx, USA).

### Ethical approval

Ethical approval was not required for this study.

## Results

### SARS-CoV-2 infection in the ICHD population

662 ICHD patients were included in the study. 188 patients (28.4%) contracted SARS-CoV-2 infection during the study period. One patient was reinfected with SARS-CoV-2 fifteen months after initial infection.

### Predictors of SARS-CoV-2 infection and mortality

Table 1 shows the baseline characteristics of the ICHD cohort by SARS-CoV-2 infection status and Table 2 shows a univariable logistic regression model demonstrating the association of variables with SARS-CoV-2 infection. Age (Odds Ratio, OR 0.97 (0.89–1.09) for every 10-year increase in age) and gender (OR 0.9 (0.64–1.3) for female) had no influence on infection rates. Compared to Caucasians, Asians had a 43% higher risk of contracting SARS-CoV- 2 infection, but this was not significant. Black and mixed/other ethnic groups had less risk of contracting SARS-CoV-2 infection but this also was not significant. There was no significant difference in SARS-CoV-2 infection rates between hub and satellite dialysis units.

**Table 1:** Baseline CHARACTERISTICS in infected vs non-infected ICHD patients

Characteristics		SARS-CoV-2 + ve *N* = 188 (28.4%)	SARS-CoV-2 -ve *N* = 474 (71.6%)	*P*-Value
**Median age** (IQR) years		65.0 (22.5)	65 (21)	0.70
**Age group**	16 to 40 yr N (%)	21 (30.9)	47 (69.1)	
	41 to 50 yr N (%)	20 (32.8)	41 (67.2)	
	51 to 60 yr N (%)	32 (25.4)	94 (74.6)	
	61 to 70 yr N (%)	43 (27.4)	114 (72.6)	
	71 to 80 yr N (%)	49 (30.1)	114 (69.9)	
	≥ 81 yr N (%)	23 (26.4)	64 (73.6)	
**Gender**	Male N (%)	113 (27.6)	297 (72.)	0.54
	Female N (%)	75 (29.8)	177 (70.2)	
**Ethnicity**	Asian N (%)	12 (36.4)	21 (66.7)	0.47
	Black N (%)	5 (20.8)	19 (83.3)	
	Mixed/Other N (%)	2 (16.7)	10 (83.3)	
	White N (%)	169 (28.5)	424 (71.5)	
**Dialysis Units**	Hub N (%)	49 (30.6)	111 (69.4)	0.47
	Satellite N (%)	139 (27.7)	363 (72.3)

**Table 2:** Univariable logistic regression analysis of risk factors

SARS-CoV-2 -ve (474) = 0 SARS-CoV-2 + ve (188) = 1		Univariable model	
		**OR (95% CI)**	***P*-value**
**Age (10-year band)**		0.97 (0.89–1.09)	0.53
**Age (years)**	16 to 40 **(ref)**	1.0	-
	41 to 50	1.1 (0.52–2.29)	0.82
	51 to 60	0.76 (0.40–1.46)	0.41
	61 to 70	0.84 (0.45–1.47)	0.59
	71 to 80	0.80 (0.52.1.78)	0.90
	≥ 81 yr	0.80 (0.40–1.62)	0.54
**Gender**	**Female (ref)**	0.90 (0.64–1.30)	0.54
	**White (ref)**	1.0	-
	**Asian**	1.43 (0.69–2.98)	0.33
**Ethnicity**	**Black**	0.66 (0.24–1.80)	0.41
	**Mixed/Other**	0.50 (0.11–2.31)	0.38
**Dialysis location**	**Satellite (ref)**	1.0	-
	**Hub**	1.15 (0.78–1.70)	0.47

Of those 188 ICHD patients who contracted SARS-CoV-2, we classified their SARS-CoV-2 infection based on the WHO clinical progression scale. Almost half of patients (48.4%) either had asymptomatic infection or ambulatory mild disease that did not require admission.

### Outcomes

Of the 188 ICHD patients who contracted SARS-Cov-2, 51.1% required hospitalisation and 19.2% died within 28 days of testing positive for SARS-CoV-2. Of the 188 infected patients, 34% had died by the end of the study period compared to 19.8% in those who had not contracted SARS-CoV-2 infection (*p* value < 0.001).

**Fig. 1:**
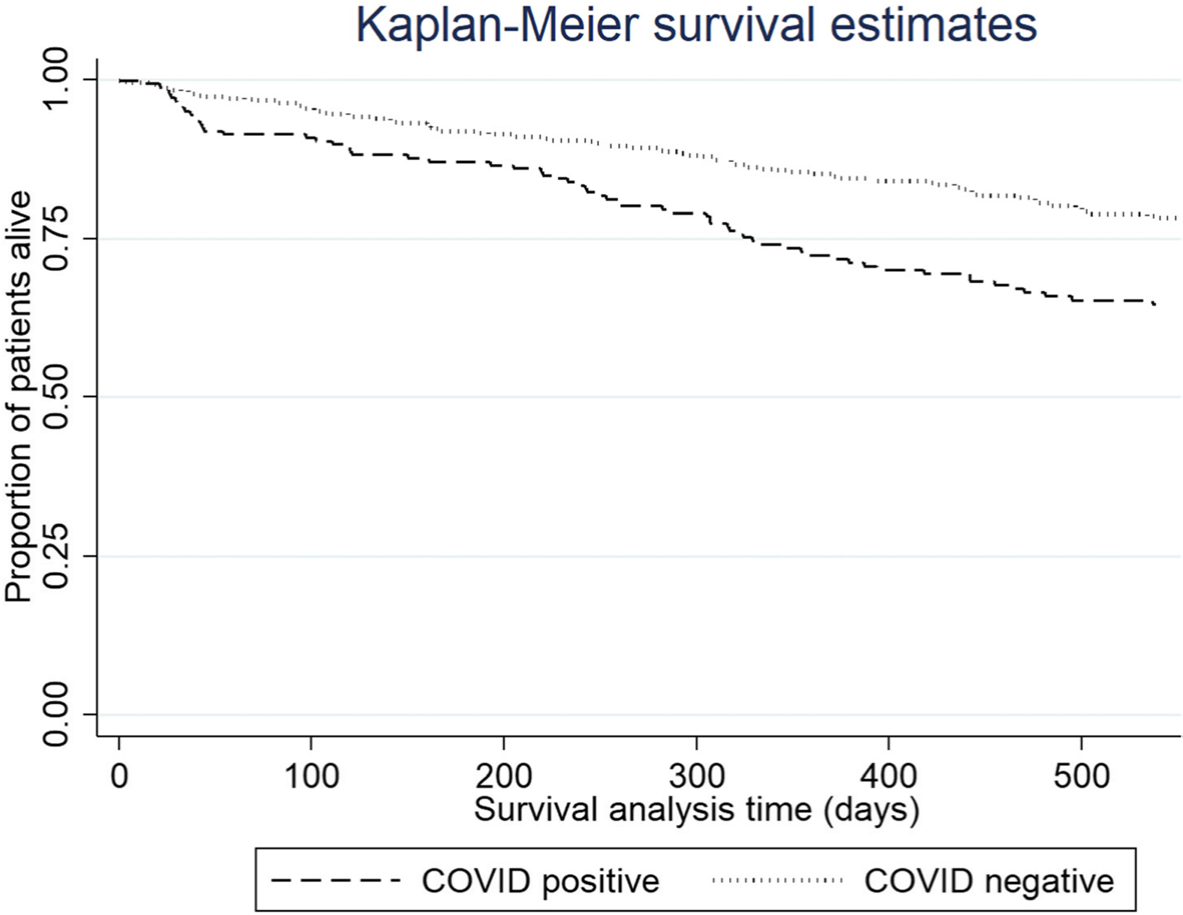
Kaplan Meier survival analysis between SARS-CoV-2 positive and SARS-CoV-2 negative patients

Figure 1 shows the unadjusted mortality at one year for the SARS-CoV-2 positive and negative cohorts. The survival curves continue to diverge at 500 days. Table 3 demonstrates the annual mortality and survival figures for the ICHD population in Liverpool for five years between 2014–2018 (UK Renal Registry Data). The average mortality over the five years was 15.5%. Therefore SARS-CoV-2 infection was an independent risk factor for mortality resulting in significant excess deaths (34.0%) over the period studied.

**Table 3:** Prevalent in-centre haemodialysis mortality 2014–2018 (UK Renal Registry Data)

Prevalent cohort	Prevalent N	Deaths N	Died %	Survival %
2014	519	77	14.8	85.2
2015	533	83	15.6	84.4
2016	540	89	16.5	83.5
2017	546	81	14.8	85.2
2018	555	88	15.9	84.1

Table 4 shows unadjusted and adjusted all-cause mortality for SARS-CoV-2 positive and SARS-CoV-2 negative patients. Patients with SARS-CoV-2 infection were 1.8 times more likely to die over the study period compared to those that remained negative (HR 1.81 (1.32–2.49) *P* < 0.001)). Mortality remained consistent despite adjustment for age, gender and ethnicity.

**Table 4:** Unadjusted and adjusted all-cause mortality, hazard ratio (HR), 95% confidence interval (CI) and (*p*-value), for SARS-CoV-2 positive and SARS-CoV-2 negative patients

		**Unadjusted Model**		**Univariable Model 1**		**Univariable Model 2**		**Univariable Model 3**			**Univariable Model 4**	
				**(Age)**		**(Gender)**		**(Ethnicity)**			**(Dialysis location)**	
**Cohort**		HR (95% CI)	*p* value	HR (95% CI)	*p* value	HR (95% CI)	*p* value		HR (95% CI)	*p* value	HR (95% CI)	*p* value
**SARS-CoV-2 –ve (*n* = 474)**		1.0	-	1.0	-	1.0	-		1.0	-	1.0	-
**SARS-CoV-2 + ve (*n* = 188)**		1.81 (1.32–2.49)	< 0.001	1.88 (1.37–2.60)	< 0.001	1.82 (1.33–2.51)	< 0.001		1.81 (1.32–2.50)	< 0.001	1.83 (1.33–2.52)	< 0.001
**Age**	**10-year bands**			1.26 (1.13–1.42)	< 0.001							
**Gender**	**Female (ref)**					1.07 (0.77–1.48)	0.68					
**Ethnicity**	**Non-Asian (ref)**								0.86 (0.41–1.85)	0.71		
**Dialysis location**	**Satellite (ref)**										2.36 (1.70–3.27)	< 0.001

### Hub vs satellite unit

As shown above in Table 1, there was no significant difference in SARS-CoV-2 infection rates between the hub units compared to the satellite units. However, 21.3% of the patients who routinely dialysed in hub units and acquired SARS-CoV-2 died within 28 days, compared to 9.4% of those who dialysed in satellite units and were infected. (*P* value 0.007).

### Comparison of SARS-CoV-2 in the ICHD population and in the community

**Fig. 2:**
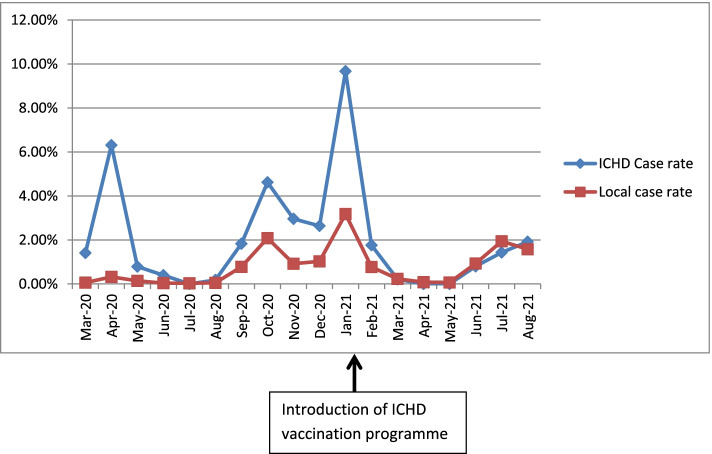
A comparison of case rates between the ICHD population and the local population

28.4% of ICHD population were infected with SARS-CoV-2 at some point within the study period, compared to 13.9% of the community. Infection rates in ICHD patients and the local population are presented in Fig. 2. The SARS-CoV-2 rates in the ICHD and local populations peaked at the same time, but a considerably higher proportion of ICHD patients contracted SARS-CoV-2.

The ICHD hospitalisation rate over the study period was 51.1% compared to 9.0% of the local population. The ICHD SARS-CoV-2 mortality rate was 19.2% and significantly higher than the 1.8% SARS-CoV-2 related deaths in the local population.

### The SARS-CoV-2 vaccination programme

517 ICHD patients received the SARS-CoV-2 vaccination, 360 patients received ChAdOx1 and 157 received the BNT162b2 vaccination. 145 patients included within the study did not receive a vaccine but is important to note 110 of these patients died by June 2021. 35 people remained unvaccinated.

**Fig. 3:**
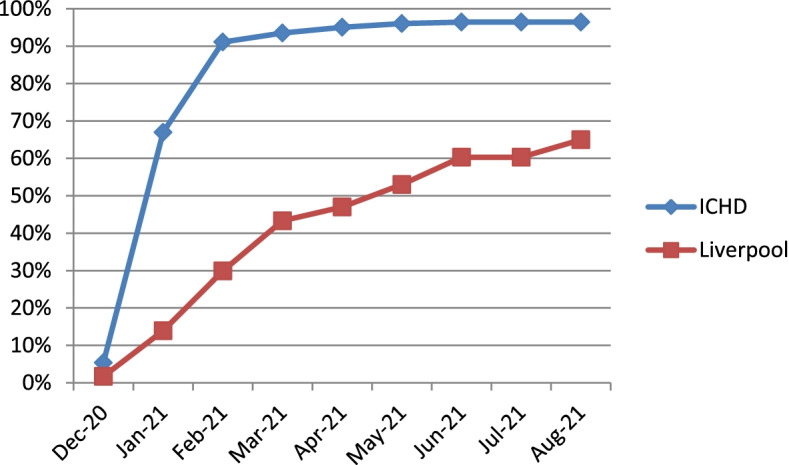
SARS-CoV-2 vaccination uptake graph in the ICHD and Liverpool population (by first dose)

**Fig. 4:**
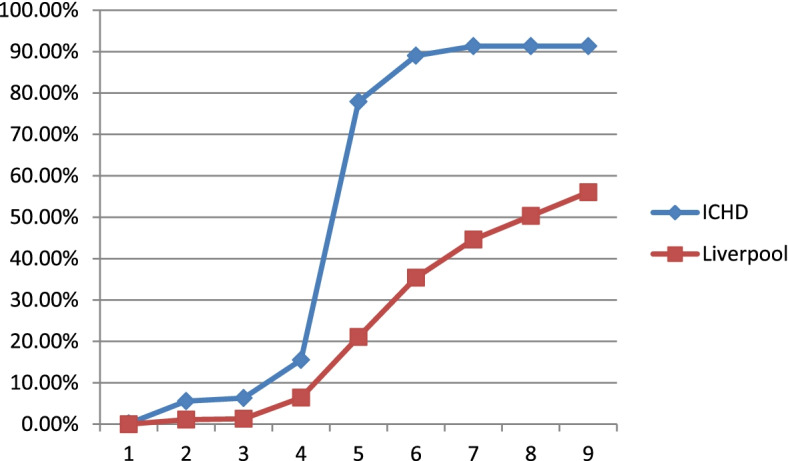
SARS-CoV-2 vaccination uptake graph in the ICHD and Liverpool population (by second dose)

Figure 3 shows that by 31^st^ August 2021 96.4% of dialysis patients had received the first vaccine. This is in contrast to only 65% of the population in Liverpool (age 12 +) had been vaccinated by the 31st August 2021 [15]. Figure 4 shows that 91.3% of ICHD patients had received the first and second SARS-CoV-2 vaccine by 31^st^ August 2021, compared to just 56% of the local population (age 12 + in Liverpool). Despite encouragement and education, a small number of patients did not receive the SARS-CoV-2 vaccination.

**Fig. 5:**
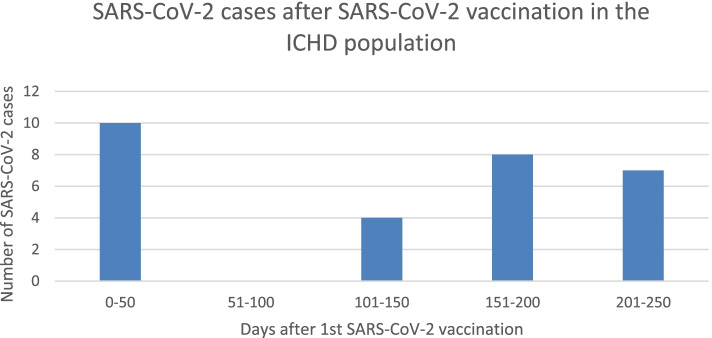
SARS-COV-2 cases after SARS-CoV-2 vaccination

Of those vaccinated, 29 patients contracted SARS-CoV-2 infection after the first dose of SARS-CoV-2 vaccination. Figure 5 shows 10 cases were within the first 100 days and there were 19 cases between day 101 and day 250. The average time interval between first and second dose of the SARS-CoV-2 vaccination were 79 days.

The average time of contracting SARS-CoV-2 was 156 days after the first dose of SARS-CoV-2 vaccination and the range was from 2 to 250 days. Of those that contracted SARS-CoV-2 after the vaccination, 75.9% (*N* = 22) had received the ChAdOx1 vaccination and 24.1% (*N* = 7) received the BNT162b2 vaccination.

Table 5 shows the SARS-CoV-2 rate ratio before and after 1^st^ dose of SARS-CoV-2 vaccine. The SARS-CoV-2 rate ratio in the post 1^st^ dose vaccination cohort was lower.

**Table 5:** SARS-CoV-2 rate ratio in the pre and post-vaccination cohort following 1st dose of SARS-CoV-2 vaccine

	**Pre 1**^**st**^** dose vaccine cohort** ***N* = 503**	**Post 1**^**st**^** dose vaccine cohort** ***N* = 499**
SARS-CoV-2 Infection (N)	159	29
Period at risk (days)	133,979	90,267
SARS-CoV-2 Rate Ratio	0.0012	0.0003

## Discussion and conclusions

Our findings have shown that SARS-CoV-2 infection is significantly more common in the ICHD population (28.4%) than the local population (13.9%). A study from Ontario found that 1.5% of dialysis patients contracted SARS-CoV-2 from 12^th^ March 2020 to 20^th^ August 2020, compared to 0.3% of those in the provincial population [18]. Similarly, they found high hospitalisation and mortality rates in their dialysis patients. 62.6% required hospital admission (compared to 51.1% in our study) and their case fatality rate was 28.3% in comparison to 19.2% in our patients. Their multivariable analysis identified independent predictors of SARS-CoV-2 infection to be in-centre haemodialysis, black, Indian subcontinent and other non-white ethnicities, lower income quintiles and those residents in long term care. Interestingly they found that age, diabetes and other comorbidities were not predictive.

In our study, age, gender and ethnicity were not independent predictors of SARS-CoV-2 infection. Asians had a 43% higher risk of contracting SARS-CoV-2 infection but this was not significant. Similarly, black and mixed/other ethnic groups had 34% and 50% less risk respectively of contracting SARS-CoV-2 infection but this was not significant. Asian, black, mixed/other ethnic groups constitute a very small proportion of the population of Merseyside, hence drawing inferences on ethnicity association of SARS-CoV-2 in this population is difficult. Unlike the Canadian cohort, infection rates were similar in our satellite and hospital dialysis patients. However, mortality was higher in patients who dialysed in hospital hubs which is not surprising as it was their comorbidities that necessitated their dialysis within a hospital setting.

As demonstrated in the results, the mortality rate in those that contracted SARS-CoV-2 was 19.2% at 28 days and increased to 34.0% over the study period. Mortality over our study period was higher than previous years ICHD mortality rates, when compared to our UK Renal Registry data, therefore confirming that there have been excess deaths. The survival curve continues to diverge at 18 months suggesting that the long term mortality in those that have previously contracted SARS-CoV-2 is likely to be increased.

The high ICHD case rates demonstrated in our study and elsewhere underline the requirement of a successful vaccination programme alongside the non-pharmacological interventions we had previously implemented [4]. The UK Renal Association highlighted this urgent need for SARS-CoV-2 vaccinations in our ICHD population [19]. Following this guidance, we immediately set up our programme as described above. Uptake of the vaccine has been high. 91.3% of ICHD patients had received both vaccines whereas in Liverpool only 56% of the population (age 12 +) had received both SARS-CoV-2 vaccinations by 31^st^ August 2021. Figure 2 shows that during the first three peaks of the SARS-CoV2 pandemic, the ICHD case rate was significantly higher than the local population but more recently, since the introduction of the SARS-CoV-2 vaccination, the ICHD rate is comparable to that of the local population.

Like our vaccine programme, other hospitals have also implemented similar programmes. A team at Imperial College Healthcare NHS trust offered approximately 1500 ICHD patients their first dose of the SARS-CoV-2 vaccine within 16 days of dialysis patients being included in the vaccine priority schedule [20].

In this study, we must consider that higher rates of SARS-CoV-2 infection amongst dialysis patients may in part be due to the higher likelihood of these patients being tested. For example, all dialysis patients have their temperature checked on arrival and are asked if they have any symptoms. A raised temperature or any suspicious symptoms resulted in a SARS-CoV-2 test being performed. Another factor to be aware of is that surveillance testing was introduced on 18^th^ January 2021. This means that asymptomatic cases before this date may not have been diagnosed, thereby skewing our results.

Despite following infection control measures and regularly weekly testing of SARS-CoV-2, our patients undoubtedly had greater exposure to SARS-CoV-2 due to the need for regular dialysis in shared rooms leading to contact with undiagnosed patients. This, in part, is an explanation why there is a higher incidence of infection in our population. However, we have been unable to quantify this. The one factor in the ICHD population that works to their advantage is that their regular attendance for dialysis facilitates opportunities for healthcare staff to promote the vaccine and administer it.

A further limitation of this study is that we did not account for other potential risk factors for acquiring SARS-CoV-2 infection including obesity, comorbidities or if resident in a long-term care facility.

While assessing SARS-CoV-2 vaccine efficacy in ICHD patients; it is vital to consider seroconversion rate in this immunocompromised cohort. Studies have highlighted that the seroconversion rate following SARS-CoV-2 infection in the dialysis group is high, [21] but IgG titres decline significantly subsequently [22, 23]. However, in many, immunity is sustained by positive SARS-CoV-2 antigen-specific T cell responses [24].

Previous studies on Influenza vaccine efficacy suggested a variable potency in CKD patients compared to the general population; in terms of induced neutralising antibodies titres and the durability of this immune response. Similarly, this could be a concern for SARS-CoV-2 vaccines. The immune mechanisms offered by SARS-CoV-2 vaccines largely remain under investigation; however, similar to immunity after infection, it is likely to include both humoral and cellular components. Both mRNA and viral-vectored vaccines are preferred over inactivated vaccines to induce balanced humoral and T cell immunity [25]. Regardless of the mechanisms involved, the reduction in infection and mortality rates are the results that will determine the efficacy of any vaccination programme and our study does demonstrate the lowering of infection rates. Further studies are needed to monitor long term post-vaccination antibody levels and T cell immunity assays to assess potency and if any of the vaccines are superior to others in our dialysis population. A study is currently underway at LUFHT to correlate these outcomes with humoral responses over a further 12 month period.

In conclusion our study reinforces the findings of others that ICHD patients are particularly vulnerable to SARS-CoV-2. The infection, hospitalisation and mortality rates are considerably higher than in the general population. ICHD infections mirrored community rates albeit at a greater magnitude. One positive factor that we have demonstrated is that with their regular attendance for dialysis, vaccine coverage can be expanded rapidly and vaccine acceptance is high. Although preliminary, the drop in infection rates after vaccination is encouraging. Therefore, vaccinations in conjunction with the non-pharmacological interventions that we have previously described [4] are likely to be the key factors that will help to protect this susceptible population. All the more so as new variants emerge and case numbers rise again in many parts of the world.

## Data Availability

The datasets used and/or analysed during the current study are not publicly available but are available from the corresponding author on reasonable request.

## Abbreviations

ICHD: In centre haemodialysis
SARS-CoV-2: Severe Acute Respiratory Syndrome Coronavirus 2
LUFHT: Liverpool University Foundation Hospital Trust
RT-PCR: Reverse transcription − polymerase chain reaction
GP: General practice
CKD: Chronic kidney disease
